# Laboratory-Confirmed Influenza-Associated Hospitalizations Among Children and Adults — Influenza Hospitalization Surveillance Network, United States, 2010–2023

**DOI:** 10.15585/mmwr.ss7706a1

**Published:** 2024-10-31

**Authors:** Angelle Naquin, Alissa O’Halloran, Dawud Ujamaa, Devi Sundaresan, Svetlana Masalovich, Charisse N. Cummings, Kameela Noah, Seema Jain, Pam Daily Kirley, Nisha B. Alden, Elizabeth Austin, James Meek, Kimberly Yousey-Hindes, Kyle Openo, Lucy Witt, Maya L. Monroe, Justin Henderson, Val Tellez Nunez, Ruth Lynfield, Melissa McMahon, Yomei P. Shaw, Caroline McCahon, Nancy Spina, Kerianne Engesser, Brenda L. Tesini, Maria A. Gaitan, Eli Shiltz, Krista Lung, Melissa Sutton, M. Andraya Hendrick, William Schaffner, H. Keipp Talbot, Andrea George, Hafsa Zahid, Carrie Reed, Shikha Garg, Catherine H. Bozio

**Affiliations:** ^1^CDC, Atlanta, Georgia; ^2^Goldbelt Inc., Chesapeake, Virginia; ^3^Oak Ridge Institute for Science and Education, Oak Ridge, Tennessee; ^4^California Department of Health, Richmond, California; ^5^California Emerging Infections Program, Oakland, California; ^6^Colorado Department of Public Health and Environment, Denver, Colorado; ^7^Connecticut Emerging Infections Program, Yale School of Public Health, New Haven, Connecticut; ^8^Division of Infectious Diseases, Emory University School of Medicine, Atlanta, Georgia; ^9^Georgia Emerging Infections Program, Georgia Department of Public Health, Atlanta, Georgia; ^10^Research, Atlanta Veterans Affairs Medical Center, Decatur, Georgia; ^11^Maryland Department of Health, Baltimore, Maryland; ^12^Michigan Department of Health and Human Services, Lansing, Michigan; ^13^Minnesota Department of Health, St. Paul, Minnesota; ^14^New Mexico Department of Health, Santa Fe, New Mexico; ^15^New York State Department of Health, Albany, New York; ^16^University of Rochester Medical Center, School of Medicine and Dentistry, Rochester, New York; ^17^Ohio Department of Health, Columbus, Ohio; ^18^Public Health Division, Oregon Health Authority, Portland, Oregon; ^19^Vanderbilt University Medical Center, Nashville, Tennessee; ^20^Salt Lake County Health Department, Salt Lake City, Utah

## Abstract

**Problem/Condition:**

Seasonal influenza accounts for 9.3 million–41 million illnesses, 100,000–710,000 hospitalizations, and 4,900–51,000 deaths annually in the United States. Since 2003, the Influenza Hospitalization Surveillance Network (FluSurv-NET) has been conducting population-based surveillance for laboratory-confirmed influenza-associated hospitalizations in the United States, including weekly rate estimations and descriptions of clinical characteristics and outcomes for hospitalized patients. However, a comprehensive summary of trends in hospitalization rates and clinical data collected from the surveillance platform has not been available.

**Reporting Period:**

2010–11 through 2022–23 influenza seasons.

**Description of System:**

FluSurv-NET conducts population-based surveillance for laboratory-confirmed influenza-associated hospitalizations among children and adults. During the reporting period, the surveillance network included 13–16 participating sites each influenza season, with prespecified geographic catchment areas that covered 27 million–29 million persons and included an estimated 8.8%–9.5% of the U.S. population. A case was defined as a person residing in the catchment area within one of the participating states who had a positive influenza laboratory test result within 14 days before or at any time during their hospitalization. Each site abstracted case data from hospital medical records into a standardized case report form, with selected variables submitted to CDC on a weekly basis for rate estimations. Weekly and cumulative laboratory-confirmed influenza-associated hospitalization rates per 100,000 population were calculated for each season from 2010–11 through 2022–23 and stratified by patient age (0–4 years, 5–17 years, 18–49 years, 50–64 years, and ≥65 years), sex, race and ethnicity, influenza type, and influenza A subtype. During the 2020–21 season, only the overall influenza hospitalization rate was reported because case counts were insufficient to estimate stratified rates.

**Results:**

During the 2010–11 to 2022–23 influenza seasons, laboratory-confirmed influenza-associated hospitalization rates varied significantly across seasons. Before the COVID-19 pandemic, hospitalization rates per 100,000 population ranged from 8.7 (2011–12) to 102.9 (2017–18) and had consistent seasonality. After SARS-CoV-2 emerged, the hospitalization rate for 2020–21 was 0.8, and the rate did not return to recent prepandemic levels until 2022–23. Inconsistent seasonality also was observed during 2020–21 through 2022–23, with influenza activity being very low during 2020–21, extending later than usual during 2021–22, and occurring early during 2022–23. Molecular assays, particularly multiplex standard molecular assays, were the most common influenza test type in recent seasons, increasing from 12% during 2017–18 for both pediatric and adult cases to 43% and 55% during 2022–23 for pediatric and adult cases, respectively. During each season, adults aged ≥65 years consistently had the highest influenza-associated hospitalization rate across all age groups, followed in most seasons by children aged 0–4 years. Black or African American and American Indian or Alaska Native persons had the highest age-adjusted influenza-associated hospitalization rates across these seasons. Among patients hospitalized with influenza, the prevalence of at least one underlying medical condition increased with increasing age, ranging from 36.9% among children aged 0–4 years to 95.4% among adults aged ≥65 years. Consistently across each season, the most common underlying medical conditions among children and adolescents were asthma, neurologic disorders, and obesity. The most common underlying medical conditions among adults were hypertension, obesity, chronic metabolic disease, chronic lung disease, and cardiovascular disease. The proportion of FluSurv-NET patients with acute respiratory signs and symptoms at hospital admission decreased from 90.6% during 2018–19 to 83.2% during 2022–23. Although influenza antiviral use increased during the 2010–11 through the 2017–18 influenza seasons, it decreased from 90.2% during 2018–19 to 79.1% during 2022–23, particularly among children and adolescents. Admission to the intensive care unit, need for invasive mechanical ventilation, and in-hospital death ranged from 14.1% to 22.3%, 4.9% to 11.1%, and 2.2% to 3.5% of patients hospitalized with influenza, respectively, during the reported surveillance period.

**Interpretations:**

Influenza continues to cause severe morbidity and mortality, particularly in older adults, and disparities have persisted in racial and ethnic minority groups. Persons with underlying medical conditions represented a large proportion of patients hospitalized with influenza. Increased use of multiplex tests and other potential changes in facility-level influenza testing practices (e.g., influenza screening at all hospital admissions) could have implications for the detection of influenza infections among hospitalized patients. Antiviral use decreased in recent seasons, and explanations for the decrease should be further evaluated.

**Public Health Action:**

Continued robust influenza surveillance is critical to monitor progress in efforts to encourage antiviral treatment and improve clinical outcomes for persons hospitalized with influenza. In addition, robust influenza surveillance can potentially reduce disparities by informing efforts to increase access to preventive measures for influenza and monitoring any subsequent changes in hospitalization rates.

## Introduction

Every year, influenza viruses circulate worldwide, causing substantial illness, severe disease, and deaths. In the United States, seasonal influenza accounts for 9.3 million–41 million illnesses, 100,000–710,000 hospitalizations, and 4,900–51,000 deaths annually (*1*). Influenza surveillance programs are important for monitoring the prevalence and characteristics of persons infected with circulating influenza viruses as well as the incidence of disease in terms of outpatient visits for influenza-like illness, hospitalizations, and deaths (*2*). During the 2003–04 influenza season, the Influenza Hospitalization Surveillance Network (FluSurv-NET) was established within CDC’s Emerging Infections Program to conduct population-based surveillance for laboratory-confirmed influenza-associated hospitalizations among children and adolescents aged <18 years. During the 2005–06 season, FluSurv-NET was expanded to include adults. Population-based surveillance involves the collection, analysis, and interpretation of data in a defined catchment area (*3*). During the 2009 influenza A H1N1 pandemic, additional states supported through a cooperative agreement with the Council of State and Territorial Epidemiologists, called the Influenza Hospitalization Surveillance Project, were integrated into FluSurv-NET. The addition of these states enabled FluSurv-NET to expand the defined catchment population and improve the generalizability of network analyses and interpretations to the rest of the country.

Since 2003, FluSurv-NET has produced near-real-time surveillance data to guide seasonal and pandemic response activities, helped to address critical public health questions, supported policy recommendations and program evaluations, and served as a platform for addressing research questions (*4*). FluSurv-NET was a crucial source of data for vaccine policy and decision-making before, during, and after the 2009 influenza A H1N1 pandemic and was leveraged to establish surveillance for COVID-19–associated hospitalizations during the COVID-19 pandemic (*4*,*5*). FluSurv-NET’s core functions are to monitor weekly rates of laboratory-confirmed influenza-associated hospitalizations during the influenza season and to collect clinical data on hospitalized patients (*3*). In addition, FluSurv-NET data provide key inputs into estimating the annual incidence of influenza illnesses, hospitalizations, and deaths, along with the estimated incidence of illness averted by influenza vaccination (*6*,*7*). FluSurv-NET data also are used to classify severity for each influenza season (*8*). FluSurv-NET case report forms have been modified over time to answer various epidemiologic questions of public health relevance, including understanding and describing health disparities and the impact of influenza vaccines and antiviral treatment (*9*–*12*). This report contains a comprehensive summary of data collected by FluSurv-NET from the 2010–11 through 2022–23 influenza seasons, including trends in cumulative rates, clinical characteristics, and outcomes for patients hospitalized with laboratory-confirmed influenza virus infections.

## Methods

FluSurv-NET methods have been previously described (*4*). Briefly, a case of a laboratory-confirmed influenza-associated hospitalization was defined as hospitalization of a person residing in the surveillance catchment area with a positive influenza laboratory result from a viral culture, direct or indirect fluorescent antibody staining, rapid antigen assay, or molecular assay (rapid or standard real-time reverse transcription–polymerase chain reaction [RT-PCR] assays) within 14 days before or any time during hospitalization. Hospitalizations with admission dates of October 1–April 30 of each season are included; however, after discussion among CDC and participating sites, active surveillance was extended beyond April 30 to June 11 during the 2021–22 influenza season due to unusually late influenza activity.

FluSurv-NET sites obtained human subjects and ethics approvals from their respective state health department and academic partner institutional review boards as needed. This activity was reviewed by CDC, deemed not research, and was conducted consistent with applicable Federal law and CDC policy.*

### Laboratory Testing

Laboratory testing for influenza is clinician-driven or conducted based on facility testing practices. Data on influenza testing and test types were obtained from review of medical and laboratory records of included patients. Since the 2017–18 influenza season, annual assessments also have been consistently conducted at the beginning of each influenza season at FluSurv-NET partner laboratories (excluding commercial and state public health laboratories) to determine the influenza test types for clinical testing of hospitalized and emergency department patients during the previous and current seasons. Multiplex testing (including respiratory viral panels) was defined in the laboratory survey as standard molecular assays that can identify pathogens from at least two molecular targets in one assay.

### Study Population

Data on laboratory-confirmed influenza-associated hospitalizations among persons of all ages were analyzed from FluSurv-NET from the 2010–11 through 2022–23 influenza seasons because of the consistency of data collected during this time. During these seasons, the catchment area of FluSurv-NET included selected counties in the following U.S. states, unless otherwise noted: California, Colorado, Connecticut, Georgia, Idaho (2010–11), Iowa (2012–13, 2020–21, and 2021–22), Maryland, Michigan, Minnesota, New Mexico, New York, Ohio, Oklahoma (2010–11), Oregon, Rhode Island (2010–11 and 2012–13), Tennessee, and Utah. The catchment area covered approximately 27 million–29 million persons or 8.8%–9.5% of the U.S. population (Supplementary Figure 1, https://stacks.cdc.gov/view/cdc/162447).

### Variables, Data Collection, and Processing

#### Case Ascertainment

During each influenza season, cases are typically identified in persons within 7 days of admission; a minimum set of patient variables including county of residence, age, sex, race and ethnicity, admission date, evidence of a positive influenza test result, and date of positive test result is sent to CDC weekly for estimation of influenza-associated hospitalization rates by selected demographics and publicly reported on interactive dashboards (*13*,*14*). Evidence of a positive result includes test type, influenza type and, for influenza viruses that have been subtyped or genotyped, an A subtype (e.g., 2009 H1N1pdm09 or H3N2) or B lineage (e.g., Yamagata or Victoria). Race and ethnicity were recorded from medical records and categorized according to the U.S. Office of Management and Budget categories of American Indian or Alaska Native (AI/AN), Asian or Pacific Islander (collected as “Asian” or “Native Hawaiian/Other Pacific Islander” during 2010–11 and 2022–23), Black or African American (Black), White, and Hispanic or Latino (Hispanic). (During analysis, persons of Hispanic or Latino origin might be of any race but are categorized as Hispanic; all racial groups are non-Hispanic.) Persons of more than one race were excluded from the hospitalization rate analyses because population denominators were not available. Other nuances to race and ethnicity data collection in this system have been previously described (*10*). Trained surveillance staff members also complete a standardized case report form by conducting detailed reviews of hospital medical records to collect additional patient-level information, including acute signs and symptoms at admission, underlying medical conditions, antiviral treatment, and interventions or outcomes during hospitalization (e.g., intensive care unit [ICU] admission, mechanical ventilation, and in-hospital death) (Supplementary Figure 2, https://stacks.cdc.gov/view/cdc/162447).

Before the 2017–18 influenza season, the case report form was completed for all identified cases. The severity of the 2017–18 season yielded the highest counts and rates of influenza-associated hospitalizations to date and prompted the development of a sampling scheme stratified by age, site, and outcome for medical record abstractions to reduce data collection time and resources on surveillance staff members. Sample size calculations by age group were performed to estimate the number of cases across all sites needed to achieve reliable point estimates and desired CIs for selected variables of interest (e.g., ICU admission, mechanical ventilation, and influenza vaccination status). Sampling rates were calculated based on the sample sizes needed per age group, and all patients that died in the hospital or within 30 days after hospital discharge were sampled. Sampling percentages varied by site and season. Sampling schemes were modified each season to account for the changing number of cases per season, except for the 2020–21 and 2021–22 seasons during which all cases were sampled (Supplementary Figure 3, https://stacks.cdc.gov/view/cdc/162447). In addition, the minimum set of patient variables continued to be collected for all cases to allow for calculation of hospitalization rates stratified by age, race and ethnicity, and sex.

#### Underlying Medical Conditions

A person was considered as having an underlying medical condition if they had at least one identified preexisting medical condition within any of the following categories: asthma; chronic lung disease; chronic metabolic disease; cardiovascular disease; neurologic or neuromuscular disorders; immunocompromising conditions; obesity (among persons aged ≥2 years); pregnancy (among persons assigned female sex at birth who were of child-bearing age); renal disease; gastrointestinal or liver disease; rheumatologic, autoimmune, or inflammatory conditions; blood disorders or hemoglobinopathies; or hypertension (collected only since the 2021–22 influenza season). These medical conditions were identified from problem lists, emergency department notes, admission histories and physical examinations, hospital transfer notes (if applicable), or progress notes.

Over time, the number of underlying medical conditions within each condition category included as discrete checkboxes on the case report form has increased to become more inclusive of conditions that were commonly reported in free-text fields before the 2019–20 influenza season (Supplementary Table 1, https://stacks.cdc.gov/view/cdc/162447). A large number of conditions were added for the 2011–12 season. As a result, this report includes underlying medical conditions data collected from the 2011–12 through 2022–23 seasons to provide the most standardized comparison of proportions over time.

#### Signs and Symptoms

Acute respiratory and non-respiratory signs and symptoms at admission have been collected since the 2014–15 influenza season and were defined as those that began or worsened within 2 weeks before admission (e.g., cough or shortness of breath). Among cases with chest radiographs performed within 3 days after admission, community-acquired pneumonia was defined as chest radiograph findings of air space density or opacity, bronchopneumonia or pneumonia, consolidation, or any infiltrates, and either the presence of ≥1 *International Classification of Diseases* version 9 or 10 code for a pneumonia diagnosis (Supplementary Table 2, https://stacks.cdc.gov/view/cdc/162447) or documentation of pneumonia in the discharge summary.

#### Antiviral Treatment and Interventions

Cases were defined as receiving influenza antiviral treatment if documentation indicated starting oseltamivir phosphate, zanamivir, peramivir, or baloxavir marboxil in ambulatory or inpatient settings within 2 weeks before admission date or any time during hospitalization. Antiviral treatment was anchored on admission date to align with the window for laboratory confirmation in the case definition, and because symptom onset date was incomplete and had variable reliability. Data also were collected on interventions (e.g., need for invasive mechanical ventilation), admission to the ICU, and in-hospital death from any cause.

#### Influenza Vaccination Status

Trained surveillance staff ascertained a patient’s current season influenza vaccination status by reviewing up to four sources: 1) a hospital medical record, 2) a state immunization registry or immunization information system, 3) follow-up with an outpatient primary care provider or a long-term care facility, and 4) an interview of the patient or a proxy. A patient was considered vaccinated if documentation in at least one source indicated receipt of at least 1 dose of the current season’s influenza vaccine at least 2 weeks before a positive influenza test date. The patient was considered unvaccinated if documentation in at least one source indicated no receipt of the current season’s influenza vaccine. If the vaccination sources did not explicitly state that the patient received or did not receive an influenza vaccine, the patient was assigned an unknown vaccination status. Before the 2013–14 influenza season, sites could not separately report information for each of the four data sources. However, starting with the 2013–14 season, an algorithm was created to allow for separate entries from each of the four sources. Because of this change, trends for influenza vaccination are reported from the 2013–14 season onward.

### Weighting

To account for the case sampling design, unweighted counts and weighted proportions of influenza-associated hospitalizations with selected clinical characteristics and outcomes were reported. Sampling weights accounting for the probability of selection were applied to estimated weighted proportions and medians. To estimate variance around weighted proportions, 1,000 bootstrap replicate weights were used.

### Statistical Analysis

Weekly and cumulative laboratory-confirmed influenza-associated hospitalization rates per 100,000 population were calculated for each influenza season from 2010–11 through 2022–23 and stratified by patient age (0–4 years, 5–17 years, 18–49 years, 50–64 years, and ≥65 years), sex, race and ethnicity, influenza type, and influenza A subtype. During the 2020–21 season, only the overall influenza hospitalization rate was reported because case counts were insufficient to estimate stratified rates; thus, all other analyses in this report excluded the 2020–21 season. Hospitalization rates by race and ethnicity were adjusted for age using direct standardization. Rate ratios were estimated using White persons as the referent group.

Population denominators used for rate estimation from the 2010–11 through 2019–20 influenza seasons were obtained from the National Center for Health Statistics bridged-race population estimates of the resident population of the United States for the indicated years, by county, age, bridged race, Hispanic origin, and sex (*15*). For the 2010–11 through 2019–20 seasons, vintage postcensal estimates corresponding to each season’s start year were applied. For the 2020–21 through 2022–23 seasons, unbridged race population denominators were obtained from the U.S. Census Bureau where vintage 2020, 2021, and 2022 estimates were applied, respectively (*16*).

Among influenza A cases, subtype information was available on a subset of cases from the 2010–11 through 2022–23 influenza seasons (excluding 2020–21). Multiple imputation using chain equations was applied to impute missing subtype. Site, month of hospital admission, and age were included in the imputation model for each season. Multiple imputation and the analysis of imputed data were conducted in SAS (version 9.4; SAS Institute).

Descriptive analyses were reported for each influenza season for patients of all ages and stratified by age group. Trend analyses were performed to assess whether significant differences existed in influenza hospitalization rates, clinical characteristics, and outcomes over the 2010–11 through 2022–23 seasons. Data from the 2020–21 season were included in the trend analysis for the overall influenza hospitalization rates but not for other analyses. For assessing trends in rates, Poisson regression was used; for assessing trends in proportions (e.g., clinical characteristics and outcomes), logistic regression was used. Intraclass correlation coefficients were used to determine whether observations were correlated by participating site; if the intraclass coefficient was >2, the site was included as a cluster variable in the logistic regression. Polynomial regression with linear, quadratic, and cubic terms for time were used for model fit. Likelihood ratio tests were used for model selection to determine whether the quadratic, cubic, or both terms would remain in the model. Significance for trend tests was based on the linear trend (p<0.05). Regression analyses assumed simple random sampling; however, sensitivity analyses confirmed that variance estimation using replicate bootstrap replicate weights yielded similar results.

A sensitivity analysis was conducted on estimating antiviral use. Infectious Diseases Society of American guidelines recommend that persons who receive a positive diagnosis for influenza in the outpatient setting should ideally receive treatment within 48 hours of symptom onset, and persons who are hospitalized with suspected or confirmed influenza should receive antiviral treatment (regardless of duration of illness) (*17*). Thus, cases were limited to those patients who tested positive for influenza within 2 days before admission or any time during hospitalization and among them, the prevalence of antiviral treatment was estimated each season. All analyses were conducted in SAS (version 9.4; SAS Institute) using survey procedures.

## Results

### Laboratory-Confirmed Influenza-Associated Hospitalization Rates

**FIGURE 1:**
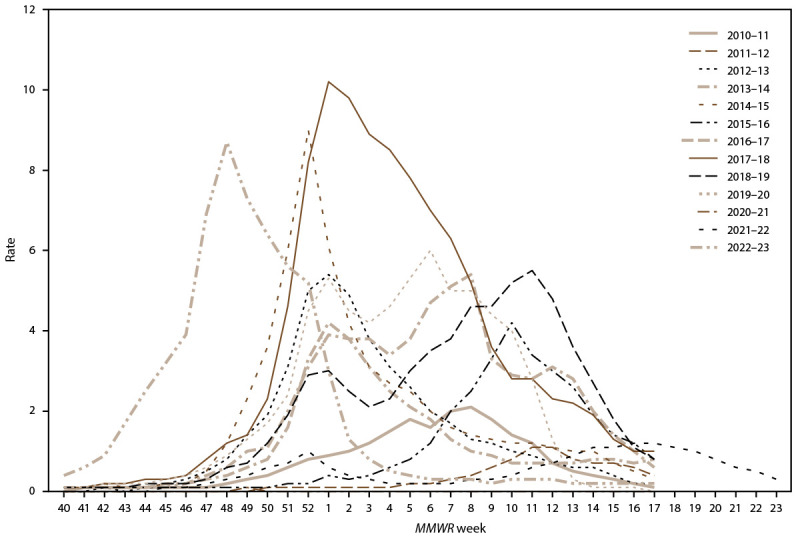
Weekly* rates^†^ of laboratory-confirmed influenza-associated hospitalizations among patients of all ages — Influenza Hospitalization Surveillance Network, United States, 2010–11 through 2022–23 influenza seasons * *MMWR* weeks correspond to approximately October (week 40) through approximately June (week 23) of the calendar year. ^†^ Hospitalization rates are reported per 100,000 population. For the 2010–11 through 2019–20 influenza seasons, U.S. population estimates were obtained from National Center for Health Statistics vintage bridged-race postcensal population estimates (https://www.cdc.gov/nchs/nvss/bridged_race/data_documentation.htm). For the 2020–21 through 2022–23 influenza seasons, unbridged race population denominators were obtained from the U.S. Census Bureau.

Among the approximately 27 million–29 million persons residing in the FluSurv-NET catchment area during the surveillance period (2010–11 through 2022–23 influenza seasons), the number of laboratory-confirmed influenza-associated hospitalization cases ranged from 2,415 during 2011–12 to 29,695 during 2017–18. Overall unadjusted hospitalization rates were determined to be statistically different across the seasons. Before the emergence of SARS-CoV-2 (the virus that causes COVID-19 disease) in 2020, influenza viruses demonstrated more consistent seasonality, with peaks occurring between late December and March (Figure 1). In addition, the magnitude of seasonal influenza-associated hospitalization rates increased before the COVID-19 pandemic, with cumulative seasonal influenza-associated hospitalization rates per 100,000 population ranging from 8.7 during 2011–12 to 102.9 during 2017–18 (Table 1). After the emergence of SARS-CoV-2, markedly different seasonal patterns emerged. The 2021–22 influenza season had a late peak in April, and the 2022–23 season peaked earlier than usual in early December (Figure 1). Influenza-associated hospitalization rates were the lowest during the 2020–21 season (0.8) and returned to recent prepandemic levels during the 2022–23 season (64.4) (Table 1).

**TABLE 1:** Laboratory-confirmed influenza-associated hospitalization rates* by season overall and by age, sex, race and ethnicity, and influenza type and subtype — Influenza Hospitalization Surveillance Network, United States, 2010–11 through 2022–23 influenza seasons

Characteristic	Influenza season												
	2010–11	2011–12	2012–13	2013–14	2014–15	2015–16	2016–17	2017–18	2018–19	2019–20	2020–21^†^	2021–22	2022–23
	Rate (95% CI)	Rate (95% CI)	Rate (95% CI)	Rate (95% CI)	Rate (95% CI)	Rate (95% CI)	Rate (95% CI)	Rate (95% CI)	Rate (95% CI)	Rate (95% CI)	Rate (95% CI)	Rate (95% CI)	Rate (95% CI)
**Age group, yrs**													
0–4	49.4 (46.3–52.5)	16.0 (14.2–17.9)	67.0 (63.2–70.7)	47.3 (44–50.5)	57.3 (53.7–60.9)	42.5 (39.5–45.6)	40.8 (37.8–43.7)	71.0 (67.0–74.9)	70.9 (67.0–74.9)	91.4 (86.9–96.0)	NA	22.4 (20.1–24.6)	87.0 (82.4–91.5)
5–17	9.1 (0.2–9.9)	4.0 (3.5–4.6)	14.6 (13.5–15.7)	9.4 (8.5–10.3)	16.6 (15.4–17.8)	9.7 (8.8–10.6)	15.5 (14.3–16.6)	19.5 (18.3–20.8)	20.0 (18.7–21.3)	23.3 (22.0–24.7)	NA	8.8 (8.0–9.7)	30.3 (28.7–31.8)
18–49	11.5 (10.9–12.0)	4.1 (3.8–4.5)	16.1 (15.4–16.8)	21.4 (20.6–22.3)	18.1 (17.4–18.9)	16.4 (15.7–17.2)	17.9 (17.1–18.6)	30.0 (29.1–31.0)	24.5 (23.7–25.4)	33.3 (32.3–34.2)	NA	9.2 (8.7–9.7)	28.2 (27.2–29.1)
50–64	22.0 (20.7–23.2)	8.1 (7.3–8.8)	40.9 (39.2–42.6)	53.7 (51.7–55.7)	53.4 (51.5–55.4)	45.2 (43.4–47.0)	62.7 (60.6–64.8)	112.8 (110.0–115.6)	79.2 (76.9–81.6)	89.4 (86.9–91.9)	NA	16.0 (15.0–17.1)	70.6 (68.4–72.8)
≥65	64.8 (62.1–67.6)	30.2 (28.3–32.1)	183.9 (179.4–188.5)	84.8 (81.7–87.9)	308.8 (303.0–314.6)	84.7 (81.8–87.7)	274.8 (269.5–280.0)	437.2 (430.7–443.7)	212.0 (207.5–216.4)	172.2 (168.3–176.2)	NA	50.8 (48.7–52.9)	185.9 (181.9–189.8)
65–74	36.9 (34.1–39.7)	17.0 (15.1–18.9)	94.3 (89.9–98.6)	68.8 (65.1–72.5)	141.2 (136.0–146.3)	66.0 (62.5–69.4)	143.1 (138.2–148.1)	245.3 (239.1–251.6)	146.4 (141.6–151.1)	135.3 (130.8–139.8)	NA	31.1 (29.0–33.2)	130.1 (125.8–134.3)
75–84	71.1 (66.0–76.2)	37.1 (33.3–40.9)	220.0 (210.8–229.2)	100.3 (94.0–106.6)	387.8 (375.5–400.0)	103.8 (97.5–110.1)	342.2 (331.0–353.5)	548.8 (534.9–562.7)	264.6 (255.2–274.0)	202.6 (194.5–210.6)	NA	70.9 (66.3–75.6)	232.9 (224.8–241.1)
≥85	156.3 (145.1–167.5)	65.7 (58.3–73.0)	468.9 (449.4–488.4)	120.0 (110.0–130.1)	874.3 (847.6–901.1)	129.0 (118.9–139.2)	741.4 (717.2–765.6)	1,117.0 (1,087.6–1,146.4)	413.7 (395.9–431.4)	286.3 (271.6–301.0)	NA	114.1 (104.4–123.8)	362.5 (345.9–379.1)
**Sex**													
Female	22.6 (21.8–23.3)	8.9 (8.4–9.3)	46.1 (45.0–47.2)	36.0 (35.0–37.0)	69.0 (67.6–70.4)	32.7 (31.8–33.7)	66.2 (64.9–67.5)	109.5 (107.8–111.2)	66.6 (65.3–67.9)	67.9 (66.6–69.2)	NA	18.8 (18.1–19.5)	69.4 (68.1–70.8)
Male	20.7 (19.9–21.4)	8.5 (8.0–9.0)	41.8 (40.8–42.9)	34.2 (33.2–35.2)	59.1 (57.8–60.4)	30.1 (29.2–31.1)	57.7 (56.4–59.0)	96.0 (94.4–97.6)	60.5 (59.2–61.7)	64.1 (62.8–65.4)	NA	16.1 (15.5–16.8)	59.2 (58.0–60.5)
**Race and ethnicity^§^**													
American Indian or Alaska Native	18.7 (13.3–24.1)	16.4 (11.1–21.7)	37.5 (29.4–45.6)	47.1 (37.8–56.4)	46.8 (37.8–55.8)	38.5 (30.1–46.9)	55.7 (45.8–65.6	57.9 (47.8–68.0)	91.7 (79.0–104.4)	96.6 (83.5–109.7)	NA	48.9 (39.0–58.8)	104.2 (89.7–118.7)
Asian or Pacific Islander	13.6 (12.1–15.1)	9.1 (7.9–10.3)	26.6 (24.6–28.6)	17.9 (16.2–19.6)	41.4 (39.0–43.8)	19.9 (18.2–21.6)	48.0 (45.5–50.5	38.3 (36.1–40.5)	36.6 (34.4–38.8)	34.1 (32.0–36.2)	NA	8.5 (7.4–9.6)	32.4 (30.3–34.5)
Black or African American	34.3 (32.6–36.0)	11.3 (10.3–12.3)	60.2 (58.0–62.4)	53.0 (50.9–55.1)	80.8 (78.4–83.2)	48.0 (46.1–49.9)	86.0 (83.5–88.5	109.4 (106.6–112.2)	91.5 (88.9–94.1)	115.8 (112.9–118.7)	NA	24.3 (22.9–25.7)	99.1 (96.3–101.9)
White	16.5 (15.9–17.1)	6.3 (5.9–6.7)	37.5 (36.5–38.5)	28.6 (27.8–29.4)	52.4 (51.2–53.6)	24.3 (23.5–25.1)	50.8 (49.6–52.0	66.1 (64.8–67.4)	53.6 (52.4–54.8)	50.9 (49.8–52.0)	NA	13.8 (13.2–14.4)	51.1 (49.9–52.3)
Hispanic or Latino	23.2 (21.8–24.6)	9.9 (9.0–10.8)	40.4 (38.6–42.2)	36.8 (35.1–38.5)	46.6 (44.8–48.4)	33.7 (32.1–35.3)	49.3 (47.5–51.1	55.5 (53.5–57.5)	65.0 (62.8–67.2)	70.8 (68.5–73.1)	NA	27.7 (26.3–29.1)	70.0 (67.7–72.3)
**Influenza type and subtype**													
Influenza A	17.8 (17.3–18.3)	7.6 (7.2–7.9)	34.9 (34.2–35.6)	31.0 (30.4–31.7)	54.8 (53.9–55.7)	23.4 (22.8–24.0)	48.5 (47.7–49.4	74.4 (73.4–75.4)	60.8 (59.9–61.7)	48.0 (47.2–48.8)	NA	17.0 (16.5–17.4)	59.5 (58.6–60.3)
A(H1N1)pdm09^¶^	6.2 (5.9–6.6)	1.6 (1.5–1.8)	1.7 (1.4–1.9)	27.3 (25.5–29.0)	0.2 (0.08–0.3)	20.6 (20.0–21.1)	1.1 (0.9–1.3	12.8 (12.1–13.5)	31.8 (31.0–32.6)	45.7 (44.9–46.5)	NA	0.04 (0.02–0.06)	15.1 (14.3–15.9)
A(H3N2)^¶^	10.8 (10.4–11.3)	5.9 (5.6–6.2)	33.2 (32.6–33.9)	3.9 (2.3–5.5)	54.9 (54.0–55.8)	2.9 (2.7–3.2)	47.7 (46.8–48.5	61.9 (60.9–63.0)	29.1 (28.3–29.9)	2.5 (2.2–2.7)	NA	16.9 (16.4–17.3)	44.3 (43.3–45.4)
Influenza B	3.6 (3.3–3.8)	1.1 (0.9–1.2)	8.9 (8.5–9.2)	3.8 (3.6–4.0)	8.8 (8.5–9.2)	7.8 (7.5–8.1)	13.1 (12.7–13.5)	27.8 (27.2–28.4)	2.5 (2.3–2.7)	17.7 (17.2–18.2)	NA	0.5 (0.4–0.6)	2.2 (2.1–2.4)
Overall**	**21.7 (21.1–22.2)**	**8.7 (8.3–9.0)**	**44.0 (43.2–44.8)**	**35.1 (34.4–35.8)**	**64.2 (63.2–65.1)**	**31.5 (30.8–32.1)**	**62.0 (61.1–62.9)**	**102.9 (101.7–104.0)**	**63.6 (62.7–64.5)**	**66.0 (65.1–67.0)**	**0.8 (0.7–0.9)**	**17.5 (17.0–18.0)**	**64.4 (63.5–65.3)**

**Abbreviation:** NA = not applicable.

* Hospitalization rates are reported per 100,000 population. For the 2010–11 through 2019–20 influenza seasons, U.S. population estimates were obtained from National Center for Health Statistics vintage bridged-race postcensal population estimates (https://www.cdc.gov/nchs/nvss/bridged_race/data_documentation.htm). For the 2020–21 through 2022–23 seasons, unbridged race population denominators were obtained from the U.S. Census Bureau.

^†^ Because of the low number of influenza-associated hospitalizations for the 2020–21 influenza season, rates and CIs stratified by age, sex, race and ethnicity, and influenza type and subtype could not be calculated.

^§^ Persons of Hispanic or Latino origin might be of any race but are categorized as Hispanic; all racial groups are non-Hispanic.

^¶^ Subtype was imputed for influenza A cases with unknown subtype using multiple imputation. The percent imputed ranged from 38% to 72% across all seasons.

** Poisson regression p value testing trend over time for overall hospitalization rate is <0.001.

Compared with other age groups, adults aged ≥65 years consistently had the highest influenza-associated hospitalization rates across all seasons, with higher rates among those aged 75–84 years and ≥85 years (Table 1). When assessing age-adjusted hospitalization rates by race and ethnicity, Black persons of all ages were hospitalized at the highest rates across most seasons, except for the 2011–12, 2018–19, 2021–22, and 2022–23 influenza seasons. During those four seasons, AI/AN persons of all ages had the highest rates. Compared with White persons, Black persons and AI/AN persons had 1.5–2.3 times and 0.9–3.5 times higher influenza-associated hospitalization rates across all seasons, respectively, and after adjusting for age.

**FIGURE 2:**
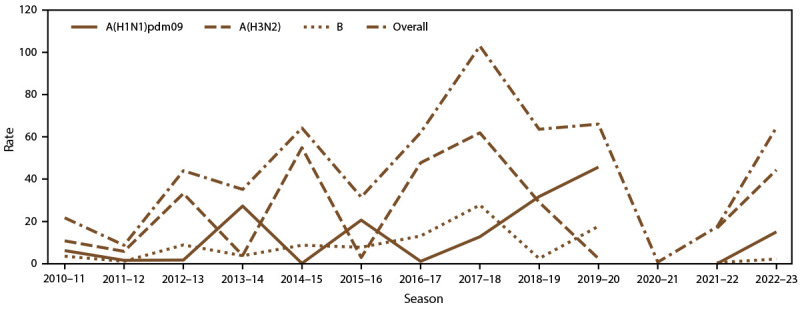
Laboratory-confirmed influenza-associated hospitalization rates* overall and by season and influenza type and subtype^†^ — Influenza Hospitalization Surveillance Network, United States, 2010–11 through 2022–23 influenza seasons^§^ * Hospitalization rates are reported per 100,000 population. For the 2010–11 through 2019–20 influenza seasons, U.S. population estimates were obtained from National Center for Health Statistics vintage bridged-race postcensal population estimates (http://www.cdc.gov/ nchs/nvss/bridged_race/data_documentation.htm). For the 2020–21 through 2022–23 influenza seasons, unbridged race population denominators were obtained from the U.S. Census Bureau. ^†^ Subtype was imputed for influenza A cases with unknown subtype using multiple imputation. The percent imputed ranged from 38% to 54% across all seasons. Poisson regression trend test p value <0.0001 for overall hospitalization rate. ^§^ Because of low case counts for the 2020–21 season, granular rates by influenza type and subtype could not be calculated.

**FIGURE 3:**
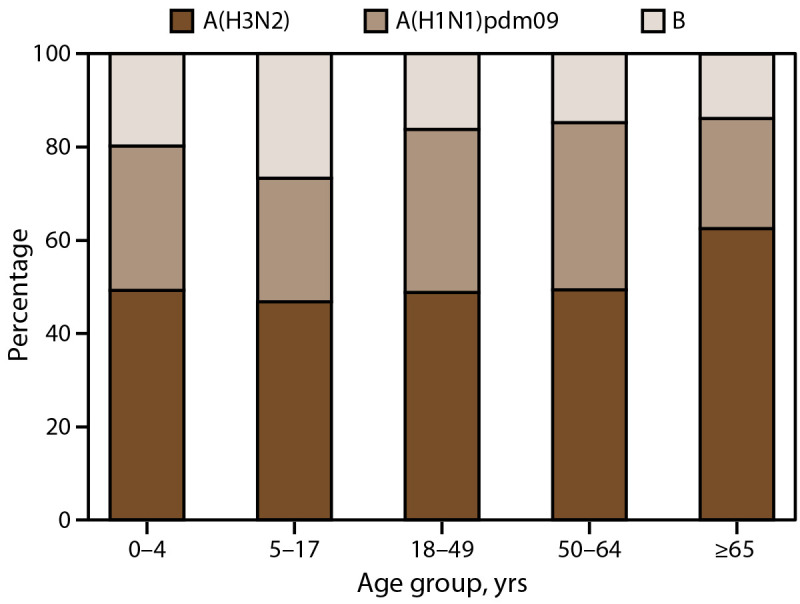
Distribution of influenza type and subtype* among laboratory-confirmed influenza-associated hospitalizations, by age group — Influenza Hospitalization Surveillance Network, United States, 2010–11 through 2022–23 influenza seasons * Subtype was imputed for influenza A cases with unknown subtype using multiple imputation. The percent imputed ranged from 38% to 72% across all seasons.

Annually, influenza A viruses caused the highest influenza-associated hospitalization rates (Table 1). After imputing for missing subtyping data, rates of hospitalizations due to influenza A(H1N1)pdm09 were higher during the 2013–14, 2015–16, 2018–19, and 2019–20 influenza seasons, whereas rates of hospitalization due to influenza A(H3N2) subtype were higher during the remaining seasons in the analytic period (Figure 2). When combining data across all seasons, influenza A(H3N2) contributed the largest proportion of cases in each age group, followed by influenza A(H1N1)pdm09 and influenza B (Figure 3). However, hospitalized children and adolescents aged 5–17 years had similar proportions of influenza B and A(H1N1)pdm09 virus infections because of the higher proportions of influenza B virus infections during the 2012–13 and 2019–20 seasons (50.1% and 55.3%, respectively) (Supplementary Table 4, https://stacks.cdc.gov/view/cdc/162447).

### Laboratory Testing for Influenza

**FIGURE 4:**
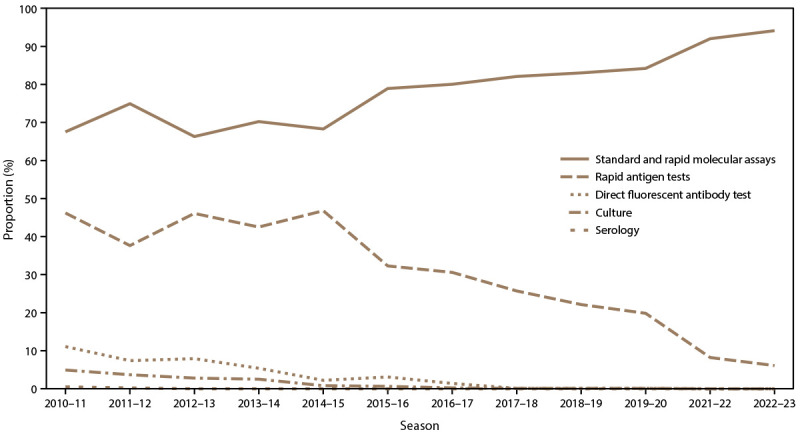
Proportions of test types*^,†^ among laboratory-confirmed influenza-associated hospitalizations with positive influenza test results, by season — Influenza Hospitalization Surveillance Network, United States, 2010–11 through 2022–23 influenza seasons **Abbreviation:** PCR = polymerase chain reaction. * Percentage of cases with this test type yielding a positive result; cases can have multiple tests and therefore test percentages are not necessarily mutually exclusive. During the 2011–12 through 2016–17 influenza seasons, 0–5 cases were tested using serology. ^†^ Rapid PCRs began being recorded during the 2016–17 influenza season, but contributed to <30% of any PCR test performed each season.

During the 2010–11 through 2022–23 influenza seasons, most (74.8%) persons hospitalized with influenza tested positive on the admission date, and 16.9% tested positive after admission (Table 2). Among persons hospitalized with influenza, molecular assays were consistently the most common influenza test type across all seasons. Notably, the proportion of molecular assays increased from 67.5% during 2010–11 to 94.1% during 2022–23 (Figure 4); rapid molecular assays specifically contributed 23% of all molecular assays performed during the 2016–17 through 2022–23 seasons when they were available. Rapid antigen tests were the second most common test type, although their use decreased over time from 46.2% during 2010–11 to 6.1% during 2022–23. All other test types accounted for <12.0% of laboratory testing among cases during 2010–11 and decreased to 0% during 2022–23.

**TABLE 2:** Timing of positive influenza test results relative to hospital admission date — Influenza Hospital Surveillance Network, United States, 2010–11 through 2022–23 influenza seasons

Timing	No. (%)
Tested positive ≥3 days before admission	1,485 (1.3%)
Tested positive 1–2 days before admission	7,638 (7.0%)
Tested positive on the same day as admission	82,499 (74.8%)
Tested positive after admission date	19,778 (16.9%)

**FIGURE 5:**
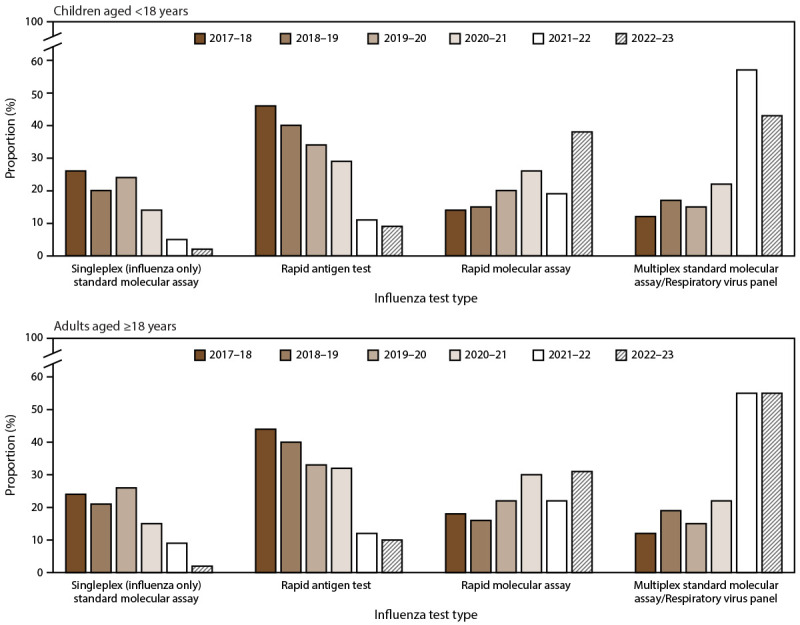
Trends of most frequently performed influenza test types* in Influenza Hospitalization Surveillance Network hospitals among children aged <18 years and adults aged ≥18 years — United States, 2017–18 through 2022–23 influenza seasons^†^ * Rapid molecular assays are a type of molecular assay that provides test results in <30 minutes. ^†^ As reported from an annual assessment of hospital laboratories that serve hospitals contributing data to the Influenza Hospitalization Surveillance Network.

On the basis of results from annual laboratory surveys from the 2017–18 through 2022–23 influenza seasons, use of the rapid molecular assays and multiplex standard molecular assays/respiratory viral panels increased over time for both pediatric and adult patients (Figure 5). Rapid antigen tests and singleplex (influenza only) standard molecular assays use decreased over time for both pediatric and adult patients.

### Clinical Characteristics Among Persons Hospitalized with Influenza

**FIGURE 6:**
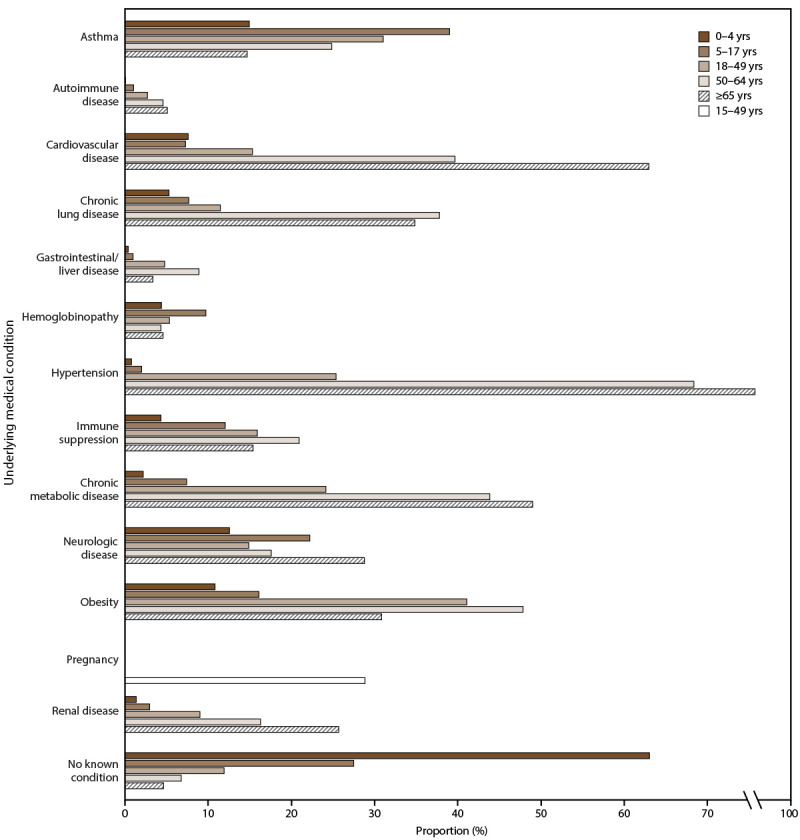
Prevalence of selected underlying medical conditions*^,†,§^ among laboratory-confirmed influenza-associated hospitalizations, by age group — Influenza Hospitalization Surveillance Network, United States, 2011–12 through 2022–23 influenza seasons * Autoimmune disease is available for the 2019–20 through 2022–23 influenza seasons. † Hypertension is available for the 2021–22 through 2022–23 influenza seasons. ^§^ Pregnancy is calculated before the 2022–23 influenza season for cases among females aged 15–44 years and during the 2022–23 influenza season for cases among females aged 15–49 years.

During the 2011–12 through 2022–23 influenza seasons, the percentage of patients within each age group with at least one underlying medical condition was 36.9% (0–4 years), 72.5% (5–17 years), 88.1% (18–49 years), 93.2% (50–64 years), and 95.4% (≥65 years) (Figure 6); these proportions were stable within each age group across all seasons. The most common underlying conditions among pediatric patients aged <18 years were asthma (0–4 years: 14.9%; 5–17 years: 39%), neurologic disorders (0–4 years: 12.6%; 5–17 years: 22.2%), and obesity (2–4 years: 10.8%; 5–17 years: 16.1%). For adult patients aged 18–49 years, the most prevalent underlying medical conditions were obesity (41%), asthma (31.1%), hypertension (25.3%), and chronic metabolic disease (24.1%). Among those aged 50–64 years and ≥65 years, the most prevalent underlying medical conditions were hypertension, obesity, chronic metabolic disease, cardiovascular disease, and chronic lung disease, although the prevalence of each condition differed between these age groups but was higher than in other age groups aged <50 years. Among persons assigned female sex at birth who were of child-bearing age and were hospitalized with influenza, 28.9% were pregnant during the 2010–11 through 2022–23 seasons.

During the 2014–15 through 2022–23 influenza seasons, most patients of all ages hospitalized with influenza reported having at least one acute respiratory sign and symptom that began or worsened within the 2 weeks before hospital admission. The proportion with at least one respiratory sign and symptom was stable during the 2014–15 through 2018–19 seasons (88.5%–90.7%) and has since decreased (82.4%–83.2%) (Table 3).

**TABLE 3:** Numbers and proportions of laboratory-confirmed influenza-associated hospitalizations of patients with any non-respiratory and respiratory sign and symptom* at admission overall and by age group — Influenza Hospitalization Surveillance Network, United States, 2014–15 through 2022–23 influenza seasons

Sign and symptom	Influenza season									
	2014–15	2015–16	2016–17	2017–18	2018–19	2019–20	2021–22	2022–23	Trend test p value^†^	Overall No. (%)
	No. (%)	No. (%)	No. (%)	No. (%)	No. (%)	No. (%)	No. (%)	No. (%)		
**Any respiratory sign and symptom at admission**										
Age group, yrs										
All ages	15,706 (88.5)	7,944 (90.4)	15,860 (90.7)	19,699 (90.3)	15,056 (90.6)	6,290 (82.8)	3,224 (82.4)	6,975 (83.2)	0.0003	**90,754 (88.0)**
0–4	861 (86.4)	672 (90.7)	628 (88.2)	1,144 (91.5)	1,137 (91.6)	1,276 (90.3)	231 (86.2)	906 (88.6)	0.0024	**6,855 (89.6)**
5–17	659 (85.7)	387 (85.8)	634 (88.1)	825 (89.6)	828 (88.0)	861 (85.8)	260 (81.8)	874 (83.9)	0.11	**5,328 (86.3)**
18–49	1,933 (86.1)	1,838 (89.6)	1,986 (88.5)	3,390 (87.6)	2,837 (89.2)	1,020 (79.6)	699 (75.2)	1,211 (73.5)	0.0001	**14,914 (83.8)**
50–64	2,540 (88.5)	2,238 (91.3)	3,146 (92.2)	4,406 (91.2)	4,006 (92.0)	1,270 (83.9)	580 (84.7)	1,222 (86.1)	0.013	**19,408 (89.3)**
≥65	9,713 (89.4)	2,809 (90.8)	9,466 (91.0)	9,934 (90.6)	6,248 (90.5)	1,863 (81.8)	1,454 (84.9)	2,762 (85.0)	0.0012	**44,249 (88.9)**
**Any non-respiratory sign and symptom^§^ at admission**										
Age group, yrs										
All ages	12,019 (67.7)	6,492 (73.9)	12,402 (70.9)	15,457 (69.5)	11,984 (71.2)	5,285 (67.2)	2,324 (59.4)	5,254 (59.2)	<0.0001	**71,217 (68.0)**
0–4	788 (79.0)	648 (87.5)	616 (86.5)	1,107 (88.5)	1,096 (88.3)	1,237 (87.5)	222 (82.8)	873 (86.2)	<0.0001	**6,587 (86.3)**
5–17	603 (78.4)	402 (89.1)	610 (84.7)	788 (85.6)	819 (87.0)	828 (82.5)	226 (71.1)	817 (79.0)	<0.0001	**5,093 (82.5)**
18–49	1,564 (69.7)	1,611 (78.6)	1,723 (76.8)	2,836 (73.3)	2,438 (76.6)	863 (67.6)	525 (50.6)	949 (55.9)	<0.0001	**12,509 (69.8)**
50–64	1,935 (67.4)	1,821 (74.3)	2,504 (73.4)	3,463 (71.2)	3,143 (72.2)	1,030 (68.0)	383 (56.5)	785 (55.2)	<0.0001	**15,064 (68.5)**
≥65	7,129 (65.6)	2,010 (64.9)	6,949 (66.8)	7,263 (65.8)	4,488 (64.7)	1,327 (58.8)	968 (56.5)	1,830 (54.6)	<0.0001	**31,964 (63.5)**

* Began or worsened within 2 weeks before admission.

^†^ Logistic regression was used in trend test analysis for statistical significance. Significance for trend tests were based on the linear trend (p<0.05).

^§^ Limited to fever or chills, altered mental status, and seizures because these signs and symptoms were consistently collected during the 2014–15 through 2022–23 influenza seasons.

### Influenza Vaccination

During the 2013–14 through 2022–23 influenza seasons, hospitalized adults aged 18–49 years consistently had the lowest proportion of patients receiving the current season’s influenza vaccine compared with other age groups (range = 15.4% during 2022–23 to 31.1% during 2014–15) (Table 4). Conversely, adults aged ≥65 years hospitalized with influenza consistently had the highest proportion of current season influenza vaccine receipt (range = 40.9% during 2022–23 to 60.7% during 2018–19). For all age groups, the season with the lowest vaccination receipt among persons hospitalized with influenza was 2022–23 (overall = 29.3%). After further stratifying by presence of a selected underlying medical condition, these trends by age remained consistent, although within each age group, influenza vaccination was higher among those with the selected underlying medical conditions than among those without the selected underlying medical conditions (Supplementary Table 5, https://stacks.cdc.gov/view/cdc/162447).

**TABLE 4:** Numbers and proportions of laboratory-confirmed influenza-associated hospitalizations that had selected clinical interventions and outcomes overall and by age group — Influenza Hospital Surveillance Network, United States, 2010–11 through 2022–23 influenza seasons

Intervention and outcome	Influenza season												Beta coefficient*	Trend test p value*
	2010–11	2011–12	2012–13	2013–14	2014–15	2015–16	2016–17	2017–18	2018–19	2019–20	2021–22	2022–23		
	No. (%)	No. (%)	No. (%)	No. (%)	No. (%)	No. (%)	No. (%)	No. (%)	No. (%)	No. (%)	No. (%)	No. (%)		
**Receipt of current season’s influenza vaccine^†^**														
Age group, yrs														
All ages	NA	NA	NA	3,149 (33.7)	8,816 (50.6)	3,450 (39.9)	8,579 (49.6)	9,608 (47.2)	7,647 (48.2)	2,590 (36.0)	1,606 (41.6)	2,082 (29.3)	0.04	<0.0001
0–4	NA	NA	NA	209 (37.2)	276 (40.1)	238 (39.8)	236 (44.4)	392 (40.3)	427 (41.8)	438 (37.7)	87 (40.1)	166 (18.1)	−0.24	<0.0001
5–17	NA	NA	NA	151 (34.9)	307 (39.9)	151 (33.5)	248 (34.4)	317 (34.4)	382 (40.6)	330 (32.9)	101 (31.8)	203 (18.3)	−0.01	<0.0001
18–49	NA	NA	NA	512 (19.4)	698 (31.1)	519 (25.3)	682 (30.4)	1,024 (26.5)	950 (29.9)	288 (22.6)	213 (22.9)	253 (15.4)	−0.05	<0.0001
50–64	NA	NA	NA	863 (30.3)	1,213 (42.3)	895 (36.5)	1,423 (41.7)	1,850 (37.8)	1,731 (39.7)	477 (31.9)	245 (35.8)	287 (24.2)	−0.07	<0.0001
≥65	NA	NA	NA	1,414 (49.3)	6,322 (58.2)	1,647 (53.2)	5,990 (57.6)	6,025 (56.2)	4,157 (60.7)	1,057 (47.4)	960 (56.1)	1,173 (40.9)	−0.20	<0.0001
**Antiviral treatment^§,^** ^¶^														
Age group, yrs														
All ages	4,427 (70.2)	1,727 (71.5)	9,737 (78.4)	8,055 (83.8)	1,5196 (85.6)	7,422 (84.4)	15,453 (88.4)	19,657 (91.7)	14,862 (90.2)	6,059 (83.3)	2,945 (75.3)	5,861 (79.1)	0.22	<0.0001
0–4	506 (53.0)	160 (55.2)	869 (71.3)	657 (80.0)	783 (78.5)	580 (78.3)	563 (79.1)	1,082 (86.5)	1,047 (84.4)	1,120 (79.3)	159 (59.3)	632 (60.9)	0.50	<0.0001
5–17	255 (55.9)	108 (56.3)	485 (69.3)	354 (81.8)	621 (80.8)	336 (74.5)	559 (77.6)	776 (84.3)	774 (82.3)	764 (76.1)	179 (56.3)	617 (59.2)	0.53	<0.0001
18–49	1,148 (75.0)	390 (74.1)	1,605 (77.8)	2,269 (85.8)	1,883 (83.9)	1,753 (85.5)	1,961 (87.3)	3,501 (90.5)	2,914 (91.6)	1,027 (81.2)	668 (71.9)	1,151 (77.3)	0.08	<0.0001
50–64	882 (73.2)	313 (72.5)	1,710 (76.9)	2,365 (83.1)	2,420 (84.3)	2,108 (86.0)	2,991 (87.6)	4,381 (91.0)	3,924 (90.1)	1,257 (84.5)	538 (78.5)	1,023 (83.5)	0.03	<0.0001
≥65	1,636 (75.7)	756 (77.5)	5,068 (81.5)	2,410 (84.1)	9,489 (87.4)	2,645 (85.5)	9,379 (90.2)	9,917 (93.0)	6,203 (91.4)	1,891 (86.1)	1,401 (81.8)	2,438 (84.3)	0.05	<0.0001
Pneumonia**														
Age group, yrs														
All ages	1,098 (20.0)	540 (24.5)	2,485 (21.9)	2,677 (30.3)	3,591 (22.3)	2,320 (29.2)	3,628 (22.5)	4,498 (22.9)	3,836 (25.7)	1,604 (26.6)	604 (20.7)	1,679 (25.3)	0.21	<0.0001
0–4	119 (18.0)	35 (16.2)	190 (22.0)	174 (28.4)	155 (25.2)	148 (28.7)	100 (21.6)	220 (25.9)	226 (28.1)	244 (28.6)	25 (18.8)	192 (30.8)	0.30	0.0003
5–17	79 (24.3)	38 (25.2)	102 (19.6)	113 (32.9)	103 (20.6)	86 (29.1)	108 (22.3)	169 (27.8)	176 (27.1)	179 (28.5)	27 (15.6)	178 (29.2)	−0.04	0.4939
18–49	268 (20.6)	99 (21.1)	356 (20.0)	766 (32.3)	364 (19.6)	572 (31.6)	403 (21.1)	772 (23.6)	739 (26.9)	268 (26.4)	101 (16.8)	268 (25.3)	0.24	<0.0001
50–64	222 (19.7)	110 (26.5)	411 (19.4)	824 (30.2)	589 (21.8)	712 (30.6)	746 (22.9)	1084 (24.0)	1169 (28.3)	383 (28.1)	125 (22.1)	314 (24.8)	0.16	<0.0001
≥65	410 (19.8)	258 (27.2)	1,426 (23.6)	800 (28.9)	2,380 (22.8)	802 (26.8)	2,271 (22.7)	2,253 (22.0)	1,526 (23.6)	530 (25.0)	326 (22.6)	727 (24.5)	0.16	0.0002
**Intensive care unit admission**														
Age group, yrs														
All ages	1,128 (17.9)	3,99 (16.5)	1,956 (15.7)	2,141 (22.3)	2,661 (15.0)	1,663 (18.9)	2,751 (15.7)	3,639 (15.4)	3,047 (17.8)	1,531 (17.2)	552 (14.1)	1,403 (15.1)	−0.05	<0.0001
0–4	146 (15.3)	43 (14.8)	212 (17.4)	157 (19.1)	207 (20.8)	140 (18.9)	161 (22.6)	277 (22.1)	250 (20.1)	284 (20.1)	56 (20.9)	182 (17.1)	0.14	<0.0001
5–17	77 (16.9)	51 (26.6)	131 (18.7)	108 (24.9)	182 (23.7)	103 (22.8)	159 (22.1)	229 (24.9)	212 (22.5)	214 (21.3)	71 (22.3)	198 (18.9)	0.18	0.0027
18–49	272 (17.8)	70 (13.3)	313 (15.2)	567 (21.5)	326 (14.5)	378 (18.4)	384 (17.1)	685 (17.7)	567 (17.8)	226 (14.7)	108 (11.6)	231 (13.9)	0.06	0.0004
50–64	271 (22.5)	84 (19.4)	388 (17.5)	745 (26.2)	511 (17.8)	514 (21.0)	637 (18.7)	874 (17.4)	875 (20.1)	362 (19.2)	116 (16.9)	273 (17.8)	−0.02	0.0015
≥65	362 (16.8)	151 (15.5)	912 (14.7)	564 (19.7)	1,435 (13.2)	528 (17.1)	1,410 (13.6)	1,574 (13.2)	1,143 (15.7)	445 (15.8)	201 (11.7)	519 (13.4)	−0.16	<0.0001
**Mechanical ventilation**														
Age group, yrs														
All ages	533 (8.5)	146 (6.0)	743 (6.0)	1,068 (11.1)	1075 (6.1)	696 (7.9)	951 (5.4)	1,295 (5.4)	1,095 (6.3)	604 (6.1)	191 (4.9)	549 (5.4)	−0.002	<0.0001
0–4	63 (6.6)	12 (4.1)	63 (5.2)	51 (6.2)	56 (5.6)	34 (4.6)	37 (5.2)	71 (5.7)	65 (5.2)	73 (5.2)	13 (4.9)	38 (3.6)	−0.18	0.0573
5–17	30 (6.6)	9 (4.7)	39 (5.6)	29 (6.7)	52 (6.8)	20 (4.4)	45 (6.3)	46 (5.0)	49 (5.2)	52 (5.2)	14 (4.4)	55 (5.5)	−0.02	0.2499
18–49	138 (9.0)	30 (5.7)	132 (6.4)	325 (12.3)	134 (6.0)	191 (9.3)	134 (6.0)	237 (6.1)	229 (7.2)	104 (5.8)	35 (3.8)	97 (5.5)	0.14	<0.0001
50–64	158 (13.1)	47 (10.9)	179 (8.1)	419 (14.7)	238 (8.3)	231 (9.4)	248 (7.3)	379 (7.3)	366 (8.4)	189 (8.6)	56 (8.2)	137 (7.5)	−0.10	<0.0001
≥65	144 (6.7)	48 (4.9)	330 (5.3)	244 (8.5)	595 (5.5)	220 (7.1)	487 (4.7)	562 (4.5)	386 (5.2)	186 (5.0)	73 (4.3)	222 (4.7)	0.05	<0.0001
**Extracorporeal membrane oxygenation**														
Age group, yrs														
All ages	22 (0.3)	12 (0.5)	24 (0.2)	51 (0.5)	54 (0.3)	46 (0.5)	29 (0.2)	73 (0.3)	59 (0.3)	45 (0.5)	5 (0.1)	20 (0.2)	−0.30	0.0622
0–4	2 (0.2)	1 (0.3)	2 (0.2)	4 (0.5)	3 (0.3)	1 (0.1)	1 (0.1)	5 (0.4)	7 (0.6)	2 (0.1)	0 (—)	3 (0.2)	−0.005	0.9184
5–17	4 (0.9)	1 (0.5)	4 (0.6)	2 (0.5)	5 (0.7)	2 (0.4)	6 (0.8)	10 (1.1)	1 (0.1)	3 (0.3)	2 (0.6)	4 (0.4)	−0.06	0.1621
18–49	4 (0.3)	3 (0.6)	3 (0.1)	23 (0.9)	10 (0.4)	18 (0.9)	7 (0.3)	27 (0.7)	23 (0.7)	13 (0.5)	0 (—)	7 (0.5)	0.58	0.1584
50–64	7 (0.6)	2 (0.5)	5 (0.2)	15 (0.5)	8 (0.3)	15 (0.6)	4 (0.1)	19 (0.4)	25 (0.6)	20 (1.2)	1 (0.1)	4 (0.1)	−1.26	<0.0001
≥65	5 (0.2)	5 (0.5)	10 (0.2)	7 (0.2)	28 (0.3)	10 (0.3)	11 (0.1)	12 (0.2)	3 (0.1)	7 (0.2)	2 (0.1)	2 (0.0)	−0.13	<0.0001
**Died in hospital**														
Age group, yrs														
All ages	197 (3.1)	58 (2.4)	313 (2.5)	336 (3.5)	535 (3.0)	246 (2.8)	529 (3.0)	857 (2.9)	505 (2.7)	433 (2.7)	88 (2.2)	506 (2.8)	−0.01	0.051
0–4	3 (0.3)	0 (—)	5 (0.4)	2 (0.2)	11 (1.1)	3 (0.4)	3 (0.4)	3 (0.2)	7 (0.6)	9 (0.6)	0 (—)	5 (0.4)	0.01	0.8788
5–17	1 (0.2)	0 (—)	12 (1.7)	1 (0.2)	8 (1.0)	2 (0.4)	2 (0.3)	10 (1.1)	4 (0.4)	4 (0.4)	0 (—)	7 (0.5)	−0.06	0.1026
18–49	44 (2.9)	6 (1.1)	23 (1.1)	67 (2.5)	18 (0.8)	34 (1.7)	29 (1.3)	55 (1.4)	45 (1.4)	52 (1.4)	1 (0.1)	31 (0.9)	−0.41	<0.0001
50–64	48 (4.0)	20 (4.6)	52 (2.3)	129 (4.5)	92 (3.2)	68 (2.8)	80 (2.3)	148 (2.4)	124 (2.8)	133 (3.3)	17 (2.5)	118 (3.2)	−0.09	0.0042
≥65	101 (4.7)	32 (3.3)	221 (3.6)	137 (4.8)	406 (3.7)	139 (4.5)	415 (4.0)	641 (3.7)	325 (3.7)	235 (4.1)	70 (4.1)	345 (4.2)	0.003	0.6817
**Total sampled cases**	**6,307**	**2,415**	**12,425**	**9,608**	**17,743**	**8,790**	**17,489**	**21,600**	**16,559**	**7,531**	**3,912**	**7,675**	**—**	**—**

**Abbreviation:** NA = not applicable.

* Logistic regression was used in trend test analysis for statistical significance. Significance for trend tests were based on the linear trend (p<0.05).

^†^ During the 2010–11 through 2012–13 influenza seasons, receipt of influenza vaccination was based on whether the patient received influenza vaccine during the fall or winter of the current influenza season. Since the 2013–14 season, receipt of influenza vaccination was based on whether one of four sources (medical chart, state immunization registry, primary care provider or long-term care facility, or patient interview) indicated that the patient received the current season’s influenza vaccination accompanied by a full or partial date of vaccination. Therefore, the trend test for only receipt of influenza vaccination included the 2013–14 through 2016–17 seasons.

^§^ Cases were defined as receiving influenza antiviral treatment if documentation indicated starting oseltamivir phosphate, zanamivir, peramivir, or baloxavir marboxil in ambulatory or inpatient settings within 2 weeks before admission date or any time during hospitalization.

^¶^ Among cases reported across the 2010–11 through 2022–23 influenza seasons, 1.3% had a positive influenza result that occurred ≥3 days before admission.

** Cases were classified as having pneumonia by chest radiograph results, corresponding pneumonia *International Classification of Diseases* version 9 or 10 codes, or a diagnosis in discharge summary.

### Clinical Course and Outcomes

**FIGURE 7:**
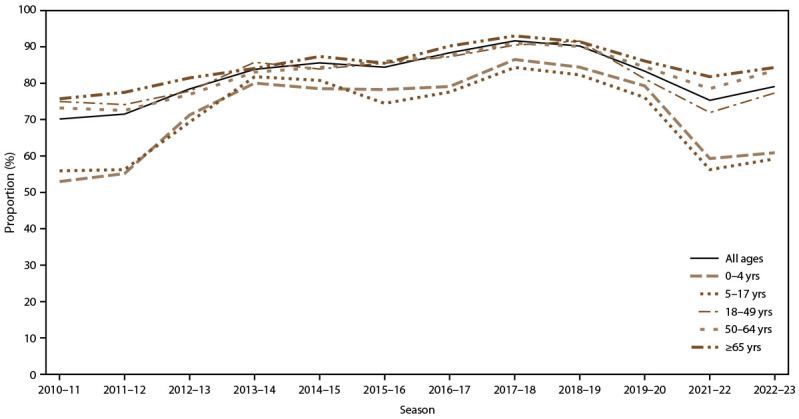
Proportions of antiviral use* among laboratory-confirmed^†^ influenza-associated hospitalizations overall and by age group — Influenza Hospitalization Surveillance Network, United States, 2010–11 through 2022–23 influenza seasons * Cases were defined as receiving influenza antiviral treatment if documentation indicated starting oseltamivir phosphate, zanamivir, peramivir, or baloxavir marboxil in ambulatory or inpatient settings within 2 weeks before admission date or any time during hospitalization. ^†^ Among cases reported across the 2010–11 through 2022–23 influenza seasons, 1.3% had a positive influenza test result that occurred ≥3 days before admission.

The proportions of patients hospitalized with influenza who received antiviral treatment increased during the 2010–11 through 2013–14 influenza seasons and remained relatively stable until the 2018–19 season (Table 4). The proportions treated across all age groups then decreased from 90.2% in 2018–19 to 79.1% in 2022–23. The decrease was more pronounced for children and adolescents aged <5 and 5–17 years; during 2018–19, the proportion who received antiviral treatment was 84.4% and 82.3%, respectively, then decreased to 60.9% and 59.2% during 2022–23, respectively. Across all seasons, children and adolescents had lower proportions of receiving antiviral treatment than adults (Figure 7). For the sensitivity analysis with a narrowed window for laboratory confirmation, 1.3% of cases were excluded because the positive test occurred >2 days before admission (Table 2). Among patients who tested positive for influenza within 2 days before admission or any time during hospitalization, the observed patterns and proportions who received antiviral treatment were similar compared with the main analysis (average percentage point difference was ±1.2% across seasons).

Among influenza-associated hospitalizations, the proportions of patients with pneumonia diagnoses ranged from 20.0% during the 2010–11 influenza season to 30.3% during the 2013–14 season, with a slightly higher proportion of pneumonia diagnoses among adults aged 50–64 years and ≥65 years compared with younger age groups (Table 4). Among persons of all ages hospitalized with influenza, the proportion admitted to the ICU ranged from 14.1% during 2021–22 to 22.3% during 2013–14. The proportion admitted to the ICU was similar across age groups within each season. Trends in mechanical ventilation use fluctuated between 4.9% during 2021–22 and 11.1% during 2013–14, whereas extracorporeal membrane oxygenation was rarely used (≤0.5% in hospitalized patients of all ages in any season). The proportion of hospitalized patients who died in the hospital ranged from 2.2% during 2021–22 to 3.5% during 2013–14, although adults, particularly those aged ≥65 years, had the highest proportions of in-hospital death (3.3% during 2011–12 to 4.8% during 2013–14).

## Discussion

Since 2003, FluSurv-NET has been a component of CDC’s influenza surveillance portfolio in the United States, serving as one of multiple surveillance systems that monitor influenza activity during each influenza season (*2*). In analyzing data from the 2010–11 through 2022–23 influenza seasons, important public health findings were observed. Adults aged ≥65 years consistently had the highest laboratory-confirmed influenza-associated hospitalization rates than other age groups. Black and AI/AN persons also had the highest age-adjusted influenza-associated hospitalization rates compared with other racial and ethnic groups. Proportions of patients who received antiviral treatment decreased since the 2018–19 season, most notably for children and adolescents, and highlight missed opportunities to prevent influenza-associated complications among those at increased risk for influenza-associated complications. The proportions of patients with underlying conditions in all age groups were similar across the seasons, underscoring that persons with selected underlying medical conditions remain at increased risk for influenza-associated complications and exacerbations of those conditions. In addition, occurrence of ICU admission, mechanical ventilation, and in-hospital death ranged from 14.1% to 22.3%, 4.9% to 11.1%, and 2.2% to 3.5% of patients hospitalized with influenza, respectively, a finding highlighting that influenza continues to cause severe morbidity and mortality. Most FluSurv-NET cases were patients with respiratory signs and symptoms at admission, although the proportion has slightly decreased over time from 90.6% during 2018–19 to 83.2% during 2022–23. Increased use of multiplex molecular assays, coupled with the decreasing proportion of hospitalized patients with acute respiratory signs and symptoms at admission, might indicate more systematic testing for respiratory viruses in recent seasons, which is important to consider when interpreting surveillance trends over time.

FluSurv-NET is one of the few CDC influenza surveillance platforms that captures data on race and ethnicity, which has been important in describing health disparities in rates of influenza-associated hospitalizations and other selected clinical outcomes. Compared with White persons, Black and AI/AN persons experienced the highest age-adjusted influenza-associated hospitalization rates and 1.4–1.8 higher age-adjusted rates of ICU admission (*10*). Lower vaccination coverage and downstream effects of individual- and neighborhood-level factors likely have contributed to the higher hospitalization rates in these groups when compared with White persons (*10*,*18*). The ability to link surveillance data with census tract data because of the network’s population-based design allows FluSurv-NET data to be used to improve understanding of these census tract–level disparities by social determinants of health and guide efforts to improve access to influenza prevention measures for racial and ethnic minorities and neighborhoods with greater social vulnerability.

The increased use of multiplex molecular assays (including respiratory virus panels), particularly since the emergence of SARS-CoV-2, coupled with the decreasing proportion of persons hospitalized with influenza with respiratory signs and symptoms at admission, might indicate that more systematic testing for respiratory viruses in hospitals is occurring regardless of clinical presentation. Consequently, more influenza infections might have been detected among persons hospitalized for reasons other than respiratory complications of their influenza infection, including exacerbations of other non-respiratory conditions. However, one challenge of the increasing use of rapid molecular assays is that most of these assays do not subtype influenza A viruses. The increasing use of these tests could further impede the ability to characterize influenza-associated hospitalizations by influenza A subtype, particularly if these subtypes differ from what is predominantly circulating within the community. These trends have been observed within laboratories serving community and academic hospitals participating in FluSurv-NET, although information on influenza testing practices, particularly after the emergence of SARS-CoV-2, beyond this surveillance system is limited.

Antiviral treatment is recommended for all hospitalized patients with suspected or confirmed influenza regardless of duration of illness (*17*). Earlier treatment initiation provides greater clinical benefit compared with late initiation (*9*,*19*,*20*), and treatment should not be delayed while laboratory results are pending. The decline in antiviral treatment of hospitalized patients with influenza from 90% during the 2018–19 influenza season to 79% during the 2022–23 season represents a concerning trend and highlights missed opportunities to prevent influenza-associated complications among those at increased risk for severe influenza complications. Declines were most notable for children and adolescents, of whom approximately 58% received antiviral treatment during the 2022–23 season. Another study observed a similar trend of increased antiviral use in pediatric influenza hospitalizations across the 2007–20 seasons, although 22% of children were not treated during hospitalization during the 2017–18 season, when antiviral treatment peaked (*21*). Antiviral treatment has been demonstrated to improve outcomes, including in-hospital survival (*22*,*23*), and both the Infectious Diseases Society of America and the American Academy of Pediatrics recommend antiviral treatment for patients of all ages hospitalized with influenza (*17*,*24*), yet treatment remains underutilized. Data are needed to identify factors associated with antiviral treatment and to better understand potential provider- and patient-level barriers, including a potential concern about adverse effects from oseltamivir use in pediatric patients (*25*). Identifying such factors could promote efforts to increase antiviral treatment among patients at highest risk for influenza-associated complications.

Influenza viruses continue to cause severe morbidity and mortality among those infected. During the analytic period, among patients hospitalized with influenza, 14.1%–22.3% were admitted to the ICU, 4.9%–11.1% were placed on mechanical ventilation, and 2.2%–3.5% died during hospitalization. The percentage of discharged patients who died after hospitalization because of their influenza infection are potentially underreported in initial FluSurv-NET surveillance; as a result, FluSurv-NET conducts post-discharge matching with death records to identify those cases. Influenza vaccines are an important tool to attenuate the morbidity and mortality associated with influenza infections and demonstrate benefits even if patients are hospitalized with influenza. Studies have demonstrated that vaccinated, hospitalized adult patients have a 26%–59% reduced risk for being admitted to the ICU and a 31% reduced risk for death compared with unvaccinated patients (*26*,*27*). According to the Advisory Committee on Immunization Practices, all persons should receive influenza vaccinations, and it is especially important for those with underlying medical conditions to be vaccinated because of the elevated risk for complications due to an influenza virus infection (*28*).

## Limitations

The findings in this report are subject to at least four limitations. First, influenza testing was clinician-driven, likely leading to underascertainment of influenza infection in seasons before the COVID-19 pandemic. To account for underdetection in surveillance, CDC employs multipliers to estimate the incidence of influenza in hospital settings nationwide; however, these multipliers were not applied in this analysis because trends in the surveillance catchment area, not nationwide, were assessed. Second, FluSurv-NET has geographically diverse sites, although the network’s catchment area covers only 8.8%–9.5% of the U.S. population. Therefore, findings might not be generalizable to all persons hospitalized with influenza in the United States. Third, selected data elements were added or changed during the analytic period; as a result, periods were restricted in assessing trends of selected characteristics to ensure consistency in seasonal comparisons. These changes reflect the natural evolution of a dynamic, adaptable, and nimble surveillance system with longstanding partners who remain vital to collecting FluSurv-NET’s robust data. Finally, multiple imputation using chain equations was performed to assign missing influenza A subtype data for 38%–54% of influenza-associated hospitalizations across all seasons.

## Conclusion

Since its establishment in 2003, FluSurv-NET has been an important platform for monitoring laboratory-confirmed influenza-associated hospitalization rates and describing the clinical epidemiology of hospitalized patients. Influenza continues to cause substantial annual morbidity and mortality, particularly among older adults, and disparities have persisted among racial and ethnic minority groups. Persons with chronic medical conditions remain at increased risk for severe influenza-associated outcomes. Antiviral use has decreased in recent seasons relative to pre–COVID-19 pandemic seasons, particularly among children and adolescents, with likely multiple contributing factors. Increased multiplex testing and other potential changes in influenza testing practices, likely in part due to the COVID-19 pandemic, could have contributed to the decrease in the proportion of hospitalized patients with laboratory-confirmed influenza with respiratory signs and symptoms at admission in recent seasons. Each of these findings are of public health importance on their own but collectively highlight the importance of conducting robust and adaptable annual public health surveillance for influenza-associated hospitalizations.

## Footnotes

* See e.g., 45 C.F.R. part 46.102(l)(2), 21 C.F.R. part 56; 42 U.S.C. Sect. 241(d); 5 U.S.C. Sect. 552a; 44 U.S.C. Sect. 3501 et seq.
